# Genetic Association of rs1021188 and DNA Methylation Signatures of *TNFSF11* in the Risk of Conductive Hearing Loss

**DOI:** 10.3389/fmed.2022.870244

**Published:** 2022-04-18

**Authors:** Amal Bouzid, Ameni Chelly, Adel Tekari, Neha Singh, Kirtal Hansdah, Imen Achour, Ikhlas Ben Ayed, Fida Jbeli, Ilhem Charfeddine, Puppala Venkat Ramchander, Rifat Hamoudi, Saber Masmoudi

**Affiliations:** ^1^Sharjah Institute for Medical Research, College of Medicine, University of Sharjah, Sharjah, United Arab Emirates; ^2^Laboratory of Molecular and Cellular Screening Processes, Centre of Biotechnology of Sfax, University of Sfax, Sfax, Tunisia; ^3^Institute of Life Sciences, Nalco Square, Bhubaneswar, India; ^4^Department of Otorhinolaryngology, Habib Bourguiba Hospital, University of Sfax, Sfax, Tunisia; ^5^Medical Genetic Department, University Hedi Chaker Hospital of Sfax, Sfax, Tunisia; ^6^Division of Surgery and Interventional Science, University College London, London, United Kingdom

**Keywords:** hearing loss, otosclerosis, bone remodeling, *TNFSF11*, rs1021188, DNA methylation

## Abstract

Otosclerosis (OTSC) is a complex bone disorder of the otic capsule, which causes conductive hearing impairment in human adults. The dysregulation of the signaling axis mediated by the receptor activator of nuclear factor-kappa-B (RANK), RANK ligand (RANKL), and osteoprotegerin has been widely attributed to the context of metabolic bone disorders. While genetic associations and epigenetic alterations in the *TNFSF11* gene (RANKL) have been well-linked to metabolic bone diseases of the skeleton, particularly osteoporosis, they have never been addressed in OTSC. This study aimed to assess whether the genetic association of rs1021188 polymorphism in the upstream of *TNFSF11* and the DNA methylation changes in its promoter CpG-region reveal the susceptibility of OTSC. Peripheral blood DNA samples were collected from unrelated Tunisian-North African subjects for genotyping (109 cases and 120 controls) and for DNA methylation analysis (40 cases and 40 controls). The gender-stratified analysis showed that the *TNFSF11* rs1021188 C/T was associated with OTSC in men (*p* = 0.023), but not in women (*p* = 0.458). Individuals with CC genotype were more susceptible to OTSC, suggesting an increased risk to disease development. Using publicly available data, the rs1021188 was within a cluster grouping the subpopulations with African ethnicity. Moreover, 26 loci in the *TNFSF11* gene were in linkage disequilibrium with rs1021188, revealing relative similarities between different populations. Significant differences in both DNA methylation and unmethylation status were detected with 4.53- and 4.83-fold decreases in the global DNA methylation levels in female and male OTSC groups, respectively. These changes could contribute to an increased risk of OTSC development. Bioinformatic analyses indicated that each of the rs1021188 variations and the DNA methylation changes in the promoter CpG-sites within *TNFSF11* may play an important role in its transcription regulation. To our knowledge, this is the first study that investigates an independent effect of the rs1021188 polymorphism and DNA hypomethylation of *TNFSF11* promoter in OTSC. Genetic and epigenetic changes in the regulatory regions of *TNFSF11* could offer new molecular insights into the understanding of the complexity of OTSC.

## Introduction

Otosclerosis (OTSC) is the primary form of conductive hearing impairment in human adults. The prevalence of this disorder ranges from 0.2 to 1% in the general population and is twice higher in women than in men (1). OTSC appears in adults with ages ranging within 30–50 years.

OTSC is histopathologically characterized by an abnormal bone growth within the otic capsule of the middle ear, giving rise to otosclerotic foci that cause stapes to oval window fixation and subsequent conductive hearing loss (2). Normally, the otic capsule undergoes very little or absent remodeling after the development and ossification of the tissue compared to the skeletal bones (3). OTSC is a complex and multifactorial disease condition caused by both environmental and genetic factors. Persistent measles virus infection can initiate pathological conditions, which, in association with other factors like autoimmunity, results in OTSC (4–6). A lot of effort has been placed in the identification of genetic causes of OTSC which resulted in the determination of 10 loci in families with autosomal dominant inheritance (7, 8). Mapping these regions failed to identify the causative gene(s) to date. Nevertheless, case-control, linkage, and association studies have evoked an eventual association of several genes with OTSC in different ethnic populations. These studies reported the possible implications of different genes, including *COL1A1, COL1A2* (9), *FGF2* (10), *TGF*β*1, BMP2* (11, 12), *BMP4* (11, 13), *AGT, ACE* (14), and *OPG* (15, 16). In addition, a genome-wide association study (GWAS) conducted in Belgian-Dutch individuals revealed that a region on chromosome 7q22.1 within the *RELN* gene is strongly associated with OTSC (17). Recent studies in OTSC families had identified six rare heterozygous variants in the *SERPINF1* gene (18), a rare heterozygous frameshift variant in the *MEPE* gene (19), and a 15 base pair coding deletion in *FOXL1* gene within a new OTSC locus of 9.96 Mb region on 16q24.1 (20).

The bone is a dynamic tissue that adapts to load variations through continuous structural remodeling for maintaining skeletal strength and integrity. Bone remodeling is a tightly regulated process, securing sequential bone resorption (removal) by osteoclasts and bone formation by osteoblasts (21). The dysregulation of these balanced processes results in metabolic bone disorders, including osteoporosis, Paget's disease, bone metastasis (22), and many other bone-related pathological conditions (23, 24). Osteoporosis has traditionally been considered an aging bone disease affecting primarily women, but elderly men also undergo substantial bone loss with age-specific hip and vertebral fractures (25, 26). On a cellular level, the disease results from osteoclastic bone resorption not compensated by osteoblastic bone formation. This leads skeletal bones to become weak, fragile and, thus, increasing the risk of fractures.

OTSC is an abnormal bone remodeling within the middle ear resulting from hypercellular and uncontrolled bone deposition in the otic capsule. The exact mechanism by which abnormal bone remodeling takes place in the disease is still not fully understood, but it is hypothesized that OTSC is triggered by bone resorption mediated by osteoclasts followed by abnormal bone apposition through osteoblasts (1). Earlier studies have been investigated the relationship and similarities between osteoporosis and OTSC. It was reported that the incidence of hearing loss was higher in patients affected with osteoporosis (27) and that, in particular, both diseases share a functionally significant polymorphism in the first intron Sp1-binding sites of the *COL1A1* gene (28).

The osteoclast-osteoblast coupling process involves different regulatory signaling macromolecules, including the receptor activator of nuclear factor-kappa-B (RANK), RANK ligand (RANKL), and the decoy receptor osteoprotegerin (OPG). Osteoclasts originate from the hematopoietic cell lineage (29, 30) and their differentiation and maturation are promoted with the binding of RANKL produced by osteoblasts to the osteoclast RANK cell surface receptor. An important antagonist in bone resorption is the OPG decoy receptor, which competitively fixes to RANKL and thereby blocks the osteoclastogenesis-mediated RANK-RANKL interaction.

An increasing number of studies suggested that the pathology of OTSC could result from a high local production of OPG in the cochlea which reduces the sensitivity of osteoclasts in the perilymphatic bone and therefore inhibit the bone turnover (31, 32). While, others concluded about regular levels and production of OPG in the otosclerotic foci (33). So far, the key molecular activators of the otic capsule remodeling remain ambiguous. Nevertheless, it is now well-established that RANKL is a critical mediator in remodeling of skeletal bone tissues and an essential osteoclasts activation factor (34–36), however, its contribution toward bone remodeling within the middle ear and the pathological initiation of OTSC has never been described.

The *TNFSF11* gene, located on human chromosome 13q14, encodes the RANKL protein. RANKL is a member of the tumor necrosis factor (TNF) cytokine family. The murine RANKL shares 83% protein sequence homology with human RANKL. The latter induces the differentiation of monocyte/ macrophage-lineage cells into the bone-resorbing osteoclasts. It consists of a C-terminal extracellular receptor-interacting domain and a transmembrane domain but is found in both membrane-bound and soluble forms (37). RANKL expression has been detected in several cell types, including lymphocytes, osteoblasts, and osteocytes (38). The contribution of RANKL in osteoclastogenesis was previously emphasized by the RANKL*-*deficient mice, which lacked lymph nodes essential for osteoclast differentiation and displayed a severe osteopetrosis phenotype characterized by radiopaque and dense long bones, vertebral bodies, and ribs (39). Only a limited number of studies explored the genetic effect of *TNFSF11* polymorphisms with bone phenotypes. A genetic study of single nucleotide polymorphisms (SNPs) in the RANK/RANKL/OPG system revealed a significant association of the rs1021188 SNP in the *TNFSF11* gene and the susceptibility of skeletal stress fracture injury in elite athletes (40). In addition, other studies provided further insight that *TNFSF11* is involved as a locus encompassing variation, including the rs1021188 SNP, to be associated with volumetric bone density and is implicated in skeletal development diseases *via* the RANK/RANKL/OPG pathway (41–43).

Given the importance of RANKL in the bone remodeling process of skeletal tissues and probably also in the otic capsule, epigenetic regulation and particularly, DNA methylation, might be involved in the bone biology of OTSC. DNA methylation is a biological process and one of the several epigenetic mechanisms that cells use to control gene expression. Methylation occurs at the cytosine nucleotides of the CpG islands present in regulatory regions and particularly in the DNA promoter region. Modifications in DNA methylation status can trigger several complex diseases, such as cardiovascular, cancer, and metabolic perturbations (44). Our earlier finding suggested that a higher CpG-site methylation level of the *CDH23* gene, expressed in the hair-cell stereocilia, is likely to be associated with age-related hearing impairment (45). It was reported that the DNA methylation status of the CpG locus three bases upstream of the TATA-box modulates the control of cell and tissue-specific expression of *TNFSF11* gene in mouse cells (46). In addition, DNA methylation was found to repress *TNFSF11* transcription which induces osteoclastogenesis *in vitro* (47), and affects the transcription expression of *RANKL-OPG* ratio indicating pathogenesis of primary osteoporosis (48).

The genetic epidemiology of variation in OTSC is not well-studied in the general population, particularly in North African populations, and requires further investigations. To this end, we aimed first to explore the association of rs1021188 SNP in the *TNFSF11* gene with OTSC in Tunisian-North African unrelated subjects. Second, we aimed to assess whether changes in DNA methylation levels within the promoter CpG-rich region of the *TNFSF11* gene reveal susceptibility to OTSC. Subsequently, bioinformatics analysis was carried out to correlate the genetics and epigenetics changes in the upstream region of the *TNFSF11* gene with its transcription regulation.

## Materials and Methods

### Patient's Selection

This study was approved by the Ethical Committee of the University Hospital of Sfax, Tunisia (CPP SUD N°28/2019), and written informed consent was obtained from all participants of this study.

The clinical and audiometric data of OTSC cases and healthy subjects were collected by our Ear, Nose and Throat specialists from the Otolaryngology Department of the University Hospital Habib Bourguiba of Sfax. OTSC diagnosis was based on audiological, clinical examinations, and surgery as previously described (49). All details of the patient characteristics were collected through a questionnaire, including demographic information, age at enrollment, detailed medical history, especially the excessive intake of drug-induced ototoxicity, otitis, the family history of hearing impairment, and metabolic/hormonal disorders. In addition, diseases deemed to affect hearing impairment, such as hypertension, diabetes, and dyslipidemia, were considered in our clinical assessment. Thus, all mentioned patient characteristics were served for the inclusion/exclusion criteria of our study population. The subject having any risk factor(s) for hearing loss was excluded from this study.

A total of 229 unrelated subjects, including 109 cases having OTSC and 120 healthy subjects, were selected for the genetic study. Genomic DNA was extracted from blood samples using a phenol-chloroform standard protocol. The same DNA was applied as a target for genotyping and quantitative methylation-specific PCR (qMSP) analyses.

### Genotype Analysis

In the present work, a cross-sectional study of the rs1021188 SNP (chr13:43116133, C > T, GRCh37/hg19) was conducted in a Tunisian North-African population. The formula *S* = [(1.96)2*p* (1− *p*)]/*d*2 was applied to calculate the sample size; where “p” is the expected prevalence in the general population based on previous studies, “d” is the absolute error. This formula estimates a type I error of 5% with a *p*-value <0.05 is statistically significant.

The rs1021188 SNP genotyping was carried out on a total of 229 unrelated subjects, on which 109 cases with OTSC (38 men, 71 women) and 120 healthy subjects (58 men, 62 women). The median age was 47 years (Interquartile Range, IQR 40–52 years) for the cases group and 60 years (IQR 51–67 years) for the control group. The rs1021188 SNP was amplified by polymerase chain reaction (PCR) followed by Sanger sequencing analysis. The forward and reverse primers of the rs1021188 SNP were designed using the Oligo-explorer software as follows: Forward: 5′-GCCTGAGAGATTGTCCCAC-3′, Reverse: 5′- GGTAAAGAAGGAAAGGCTGG-3′ and were optimized to amplify a single PCR product. PCR was carried out on an Applied Biosystems thermal cycler (Foster City, California, USA) with the following conditions: 95°C for 5 min, followed by 10 cycles of 30 s at 95°C, 30 s at 67°C, and 20 s at 72°C, and by 25 cycles of 30 s at 95°C, 30 s at 62°C and 20 s at 72°C, followed by a final extension of 5 min at 72°C. Purified PCR products were then sequenced by ABI 3500 HID Genetic Analyzer (Thermo Fisher Scientific Life, USA). Sequence chromatograms were analyzed using GeneMapper ID-X Software (Thermo Fisher).

### Quantitative Methylation-Specific PCR

For the DNA methylation analysis, a total of 80 unrelated Tunisian including 40 OTSC cases with a median age of 46 years (IQR 41–49 years) and 40 control samples with a median age of 45 years (IQR 36.5–47.5 years; equal numbers of 20 women and 20 men in each of the groups) were recruited. Sodium bisulfite treatment of the extracted genomic DNA was performed to convert the unmethylated, but not methylated, cytosines to uracil using the EZ DNA Methylation Kit (Zymo Research, Irvine, CA, USA), following the manufacturer's instructions. The DNA methylation levels were determined by qMSP (50). The PCR amplification was carried out to explore the CpG-rich regions enclosing the transcription start site (TSS) of the *TNFSF11* gene (NG_008990.1 Ref Seq Gene), precisely in the promoter regulatory region that spans from −260 to +615 bp of the TSS of the isoform 1, using the previously published primers specific for methylated and unmethylated DNA (47). For internal normalization, optimized primers of known genomic regions were used, including Human methylated DNA primer pair (*TSH2B*) (Diagenode, Liège, Belgium) for amplifying methylated DNA, and Human unmethylated DNA primer pair (*GAPDH*) (Diagenode, Liège, Belgium) for amplifying unmethylated DNA. As positive controls, we used Human bisulfite converted methylated and unmethylated DNA controls (QIAGEN, Hilden, Germany), to ensure the amplification of methylated and unmethylated products, respectively. The positive controls would also assess the DNA methylation percentage levels for both otosclerotic case and control samples. Experiments of *TNFSF11* promoter and control genes were performed in triplicate that in each a total volume of 10 μl was contained 5.5 μl Syber Premix of the Episcope MSP kit (TakaRa, Otsu, Japan), 0.5 μl of each primer pair (10 μm), and 1 μl of bisulfite-converted DNA template (10 ng/μl). Each reaction plate included *TNFSF11* and control genes with samples, and with positive controls besides water blanks. The mixture reaction was incubated for amplification and melting-curve using CFX96 Real-Time PCR detection system (Bio Rad, Redmond, WA, USA). Resulted data were interpreted using the Bio-Rad CFX Manager software (Bio Rad, Redmond, WA, USA).

### DNA Methylation Data Analysis

The quantification cycle (Cq) was reported in each reaction. Standard models of comparative threshold cycles were estimated for the methylated and unmethylated *TNFSF11* primers with positive DNA controls using OriginPro 2016 software (Northampton, Massachusetts, USA). The first set of analyses investigated the DNA methylation percentages of each sample, for both cases and controls groups, considering the methylated and unmethylated *TNFSF11* promoter primers. For this purpose, we determined the Cq values corresponding to defined ratios of methylated vs. unmethylated targets with Human bisulfite converted methylated and unmethylated DNA controls. For both groups of cases and controls, the percentage of DNA methylation levels to the total amplifiable treated DNA within the studied CpG-rich region were calculated based on the resulted standard models. To assess the difference of relative methylation levels between OTSC cases and healthy samples, the ΔΔCq method was considered using the reference genes *TSH2B* and *GAPDH*, respectively, with methylated and unmethylated *TNFSF11* promoter primers (51–53).

### Statistical Analysis

For the genotyping analysis of rs1021188 SNP, allele and genotype frequencies were determined by the direct counting method. The Hardy-Weinberg Equilibrium test was analyzed for a genotype frequency deviation in the control group with a significance level set at 0.001. The Chi-square test was used to compare the genotypes between the case and control groups. The allelic model evaluation was explored using the 2BY2 program by Fisher's exact test (54) at a significance *p*-value level of 0.05. Analysis of multivariate logistic regression was performed to estimate associations between the resulting genotypes of rs1021188 SNP and gender. The statistical analysis was carried out using the IBM SPSS Statistics software (version 23; IBM Corp, Armonk, NY, USA). A cut-off of 0.05 was considered significant for two-tailed *p*-values (*p*).

Statistical evaluations of DNA methylation levels were carried out by considering the mean of each triplicate value of *TNFSF11* promoter and reference genes. Differences in methylation and unmethylation levels between OTSC cases and controls were determined using the non-parametric Mann-Whitney test. Statistical analysis was carried out using GraphPad Prism (software version 5.0 for Windows, La Jolla, CA, USA). A *p* value <0.05 was considered statistically significant.

### Unsupervised Machine Learning Using Hierarchical Clustering

Multivariate analysis was performed using an in-house R script that applies two ways unsupervised hierarchical clustering using Euclidean and Ward's linkage method to explore the possible grouping of rs1021188 SNP distribution based on the minor allele frequency in different subpopulations and its possible association with other SNPs to be in linkage disequilibrium across several ethnic populations.

### *In silico* Analysis

The function of the rs1021188 SNP was predicted using different databases; the Regulome DB online database was explored to identify DNA features and regulatory elements in non-coding regions (https://regulomedb.org/regulome-search), HaploReg database v4.1 was carried out to annotate non-coding variants and to predict the effect of SNPs on regulatory motifs (https://pubs.broadinstitute.org/mammals/haploreg/haploreg.php), and ViennaRNA Web Servers was used to RNA secondary structures (http://rna.tbi.univie.ac.at/).

The CpG island sequence conservation for *TNFSF11* promoter was evaluated using tools provided by the University of California, Santa Cruz Genome Browser interface (GRCh37/hg19) assembly (https://genome.ucsc.edu/). Multiple alignments of 100 vertebrate species and measurements of evolutionary conservation were carried out using the phastCons and phyloP programs (55). The classification of the *TNFSF11* CpG-rich region according to their evolutionary dynamics was assessed using a parameter-rich evolutionary model and clustering analysis. Chromatin non-condensed DNaseI Hypersensitivity sites in 125 cell types were evaluated from the Encyclopedia of DNA Elements project (V3). Transcription factor binding sites were predicted using the ORegAnno database and the JASPAR Transcription Factor Binding Site database, JASPAR CORE 2022 (https://jaspar.genereg.net/).

## Results

### Genetic Association of the *TNFSF11* rs1021188 SNP With OTSC

The association of the rs1021188 polymorphism, located at the chr13:43116133 C > T within the *TNFSF11* upstream region, was evaluated for the risk with OTSC in an unrelated Tunisian-North African population composed of 109 OTSC cases and 120 healthy control subjects (controls). The rs1021188 variant was within the Hardy-Weinberg Equilibrium in the control group. Sanger sequencing revealed three genotypes distributed in the general control population: homozygous CC genotype (8.33% CC and 69.17% TT, Table 1), and heterozygous (22.5% CT). The allelic frequencies accounted for 19.6% C and 80.4% T, which were in line with the allelic frequency distributions of different observed populations in the SNP database. The minor allele frequencies accounted for 0.248 in otosclerotic cases and 0.196 in controls. The association analysis revealed no genetic statistical differences in the genotypic (*p* = 0.145) nor the allelic (*p* = 0.21) frequencies between OTSC cases and healthy controls for the rs1021188 SNP. The gender-stratified analysis between the rs1021188 SNP and OTSC, however, revealed significant genotypic and allelic associations in men (*p* = 0.023) and (*p* = 0.008), respectively, but not in women (*p* = 0.458) and (*p* = 0.2), respectively. We found an increased CC genotype in cases compared to controls in men suggesting an increased risk of OTSC.

**Table 1:** Genotype and allele frequencies of the rs1021188 SNP for otosclerotic cases and healthy controls in the Tunisian population.

**SNP**	**Genotype**	**Frequency (%)**			**Frequency (%)**			**Frequency (%)**		
	**Allele**	**Total population**			**Male**			**Female**		
		**Cases**	**Controls**	***p***	**Cases**	**Controls**	***P***	**Cases**	**Controls**	***p***
• rs1021188 • g.43116133C> T	C/C	12.8	8.3	0.145	18.4	6.9	0.023*	9.9	9.7	0.458
	C/T	23.9	22.5		28.9	20.7		21.1	24.2	
	T/T	63.3	69.2		52.6	72.4		69.0	66.1	
	C	24.7	19.6	0.21	32.9	17.2	0.008*	16.3	21.8	0.2
	T	75.3	80.4		67.1	82.8		83.7	78.2	

*Significant association (p <0.05). Bold p-values indicate significance.

### Association of the rs1021188 SNP With African Ethnicity

**Figure 1:**
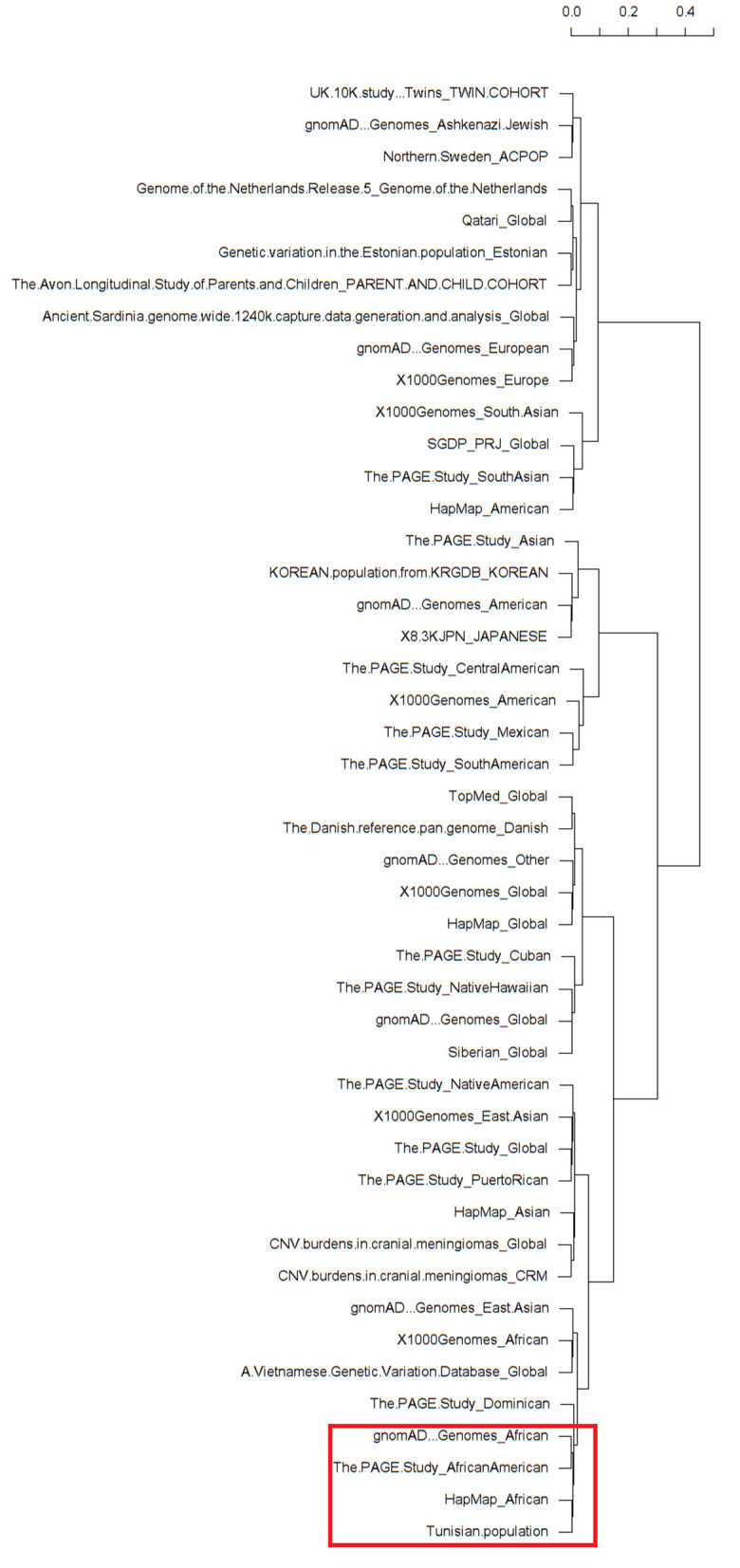
Dendrograms of a heatmap of the rs1021188 single nucleotide polymorphisms (SNP) across different ethnic groups showing subpopulation clusters. The red frame shows the related African populations including our North-African Tunisian subpopulation. The different populations that correspond to the row names showed on the dendrogram are listed in Supplementary Table.

The clustering analysis differentiated all available ethnic groups reported in the dbSNP database based on the minor allele frequency of the rs1021188 SNP (Supplementary Table). Almost all populations fall into respective clades. Interestingly, as expected, the clustering shows clear cluster grouping of the subpopulations with African ethnicity across different databases that are closely related including gnomAD – Genomes_African, The PAGE Study_African American, HapMap_African, and our North-African Tunisian population (Figure 1). Hence, the clustering information validates the association of rs1021188 SNP along with our population of African ethnicity. Moreover, the clustering analysis shows that the Dominican population next to the East Asian populations, including the Vietnamese subpopulation, fall in the same cluster with the African suggesting a similar genetic link may be due to the effect of migration and/or genetic admixture.

### Linkage Disequilibrium of rs1021188 SNP in Populations

**Figure 2:**
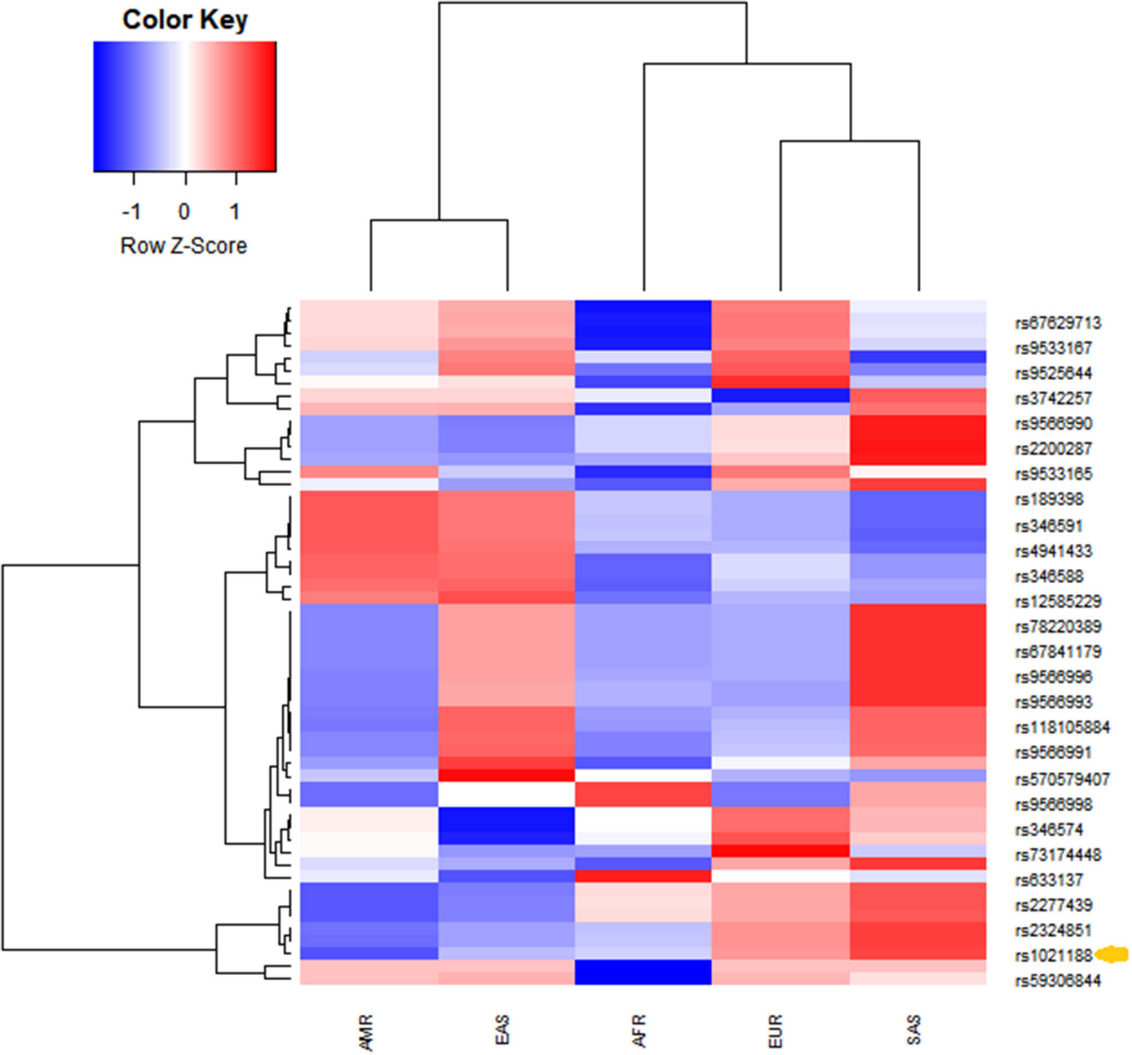
Unsupervised hierarchical clustering and heatmaps of (rows) the identified *TNFSF11* SNPs in linkage disequilibrium with the rs1021188 SNP across (columns) different populations (from the combined 1,000 Genomes project Phase3 database). Cells are color-coded according to the log10 of *p*-value, which evaluates the higher (enrichment)/lower (depletion) effect allele in a population, compared with the average of wholly one. The red color signifies a high level of allele frequency, and the blue signifies a low level of allele frequency. AFR, African (1,000 Genomes); AMR, American (1,000 Genomes); EAS, East Asian (1,000 Genomes); EUR, Europe (1,000 Genomes); SAS, South Asian (1,000 Genomes). The arrow indicates the rs1021188 SNP associated with OTSC.

The analysis of linkage disequilibrium was performed using publicly available data through the 1,000 Genomes project Phase3 Continental populations (GRCh37/hg19). SNPs in the coding sequence of *TNFSF11* with minor allele frequency > 0 were extracted. Linkage disequilibrium among rs1021188 SNP and the resulting *TNFSF11* SNPs was calculated across different populations using a phyton script. r^2^ represents the correlation between a pair of loci. The *r*^2^ value of 0 denotes that the two loci are in complete linkage equilibrium and a value of 1 represents the two loci are in complete linkage disequilibrium and coinherited. rs1021188 falls within a region transmitted in the Caucasian European population (*n* = 1,006). Twenty-six loci were in linkage disequilibrium (LD) with rs1021188 (Figure 2). Populations exhibited unique allele enrichment/ depletion patterns. Similarly, the south Asian and east Asian subpopulations show close patterns (within the same continental group). However, the SNPs in the Caucasian populations are closer to south Asians making a strong cluster and highly distinguished from the American population. African population showed a distinguished lower effect allele with depletion patterns different to Europeans. Analyzing the allele frequency for rs1021188 and *TNFSF11* SNPs alleles shows the relative closeness between different populations. Interestingly, rs1021188 and rs2324851 showed similar allele enrichment/ depletion patterns across all populations suggesting an interactive role among these two SNPs. It has been reported that rs2324851 is also significantly associated with BMD (56). Probably, the haplotype of rs1021188 (C > T) and rs2324851 (A > G) could present a genetic risk factor toward OTSC.

### The rs1021188 SNP May Affect *TNFSF11* Transcription

The *in-silico* prediction function of the non-coding variant rs1021188 in the upstream region of the *TNFSF11* gene showed that its genotype had a RegulomeDB rank of 5, computed based on the integration of multiple high-throughput datasets from Gene Expression Omnibus, the ENCODE project, and published literature. This is suggesting its probable functions as transcription factor binding or DNase peak (Supplementary Figure 1). Based on the HaploReg database, rs1021188 coincides with DNase sites and 11 altered motifs, including CEBPA, CEBPB, Dbx1, DIx3, DIx5, HNF1, Hoxb6, NKx2, Pou2f2, Prrx2, and p300. Furthermore, the influence of the rs1021188 SNP on the *TNFSF11* mRNA secondary structure was explored using the RNAfold web server. Interestingly, the rs1021188 SNP (C/T) showed local structure changes with a difference in the minimum free energy of −17.90 kcal/mol for rs1021188-C vs. −15.60 kcal/mol for rs1021188-T. Moreover, the thermodynamic ensemble diversity was different between rs1021188-C and rs1021188-T with 25.08 and 35.02, respectively, suggesting that this upstream variant may affect *TNFSF11* transcription (Supplementary Figure 2).

### CpG-Rich Region Within the *TNFSF11* Promoter

To investigate *TNFSF11* DNA methylation changes in OTSC, we evaluated a CpG-rich region within the *TNFSF11* promoter, at genomic position chr13:43148278-43149282 (GRCh37/hg19), in both otosclerotic and healthy samples. This CpG island, spanning from −260 to +615 bp of the TSS of the isoform 1 *TNFSF11* gene, shows GC content of 66.9% and a ratio of observed to expected CpG of 0.74. Using the phastCons and phyloP programs, this *TNFSF11* promoter CpG region was predicted to be conserved but in the presence of fast-evolving sites with vertebrate multiz alignment and conservation for 100 species (mean phyloP100wayAll score = 0.253015). For both otosclerotic and healthy samples, we carried out qMSP in the CpG-rich region on which methylation status was distinguished by the corresponding specific sequence primers. Deviation in the melting temperature for DNA methylated and unmethylated *TNFSF11* promoter primers, Tm = 78.5–84.5°C and Tm = 77–81.5°C, respectively, suggest that there are weak inter-individual methylation differences in the evaluated CpG-site within the *TNFSF11* promoter.

### Standard Non-Linear Models Estimating the Differentially DNA Methylation in CpG Sites of the *TNFSF11* Promoter

**Figure 3:**
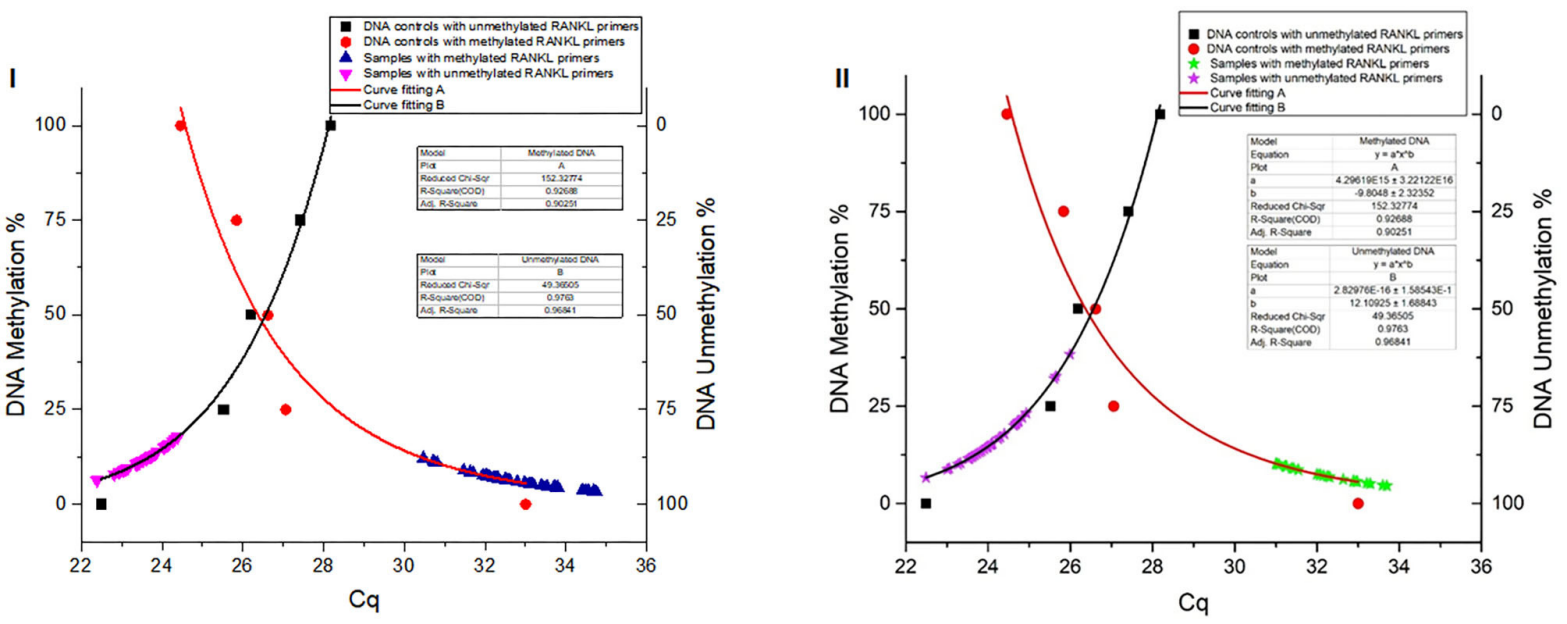
Global DNA methylation percentages of the *TNFSF11* promoter CpG-rich region in women **(I)** and men **(II)** groups using Standard non-linear Models. Curve fitting plots for serial DNA methylation percentages of converted Human DNA controls at 0, 25, 50, 75, and 100%, red and black shapes respectively, with (A) methylated *TNFSF11* primers and (B) unmethylated *TNFSF11* primers. The curve fitting plot (A) shows a proportionate increasing percentage of the methylated DNA with a decreasing of the Cq values for the *TNFSF11* methylated primers' reactions. The blue (A.I) and green (A.II) shapes illustrate resulted in DNA methylation percentages among case and control samples of quantitative amplifications with *TNFSF11* methylated primers in women and men, respectively. The curve-fitting plot (B) shows a proportionate increasing percentage of the unmethylated DNA with decreasing of the Cq values for the *TNFSF11* unmethylated primers' reactions. The pink (B.I) and purple (B.II) shapes illustrate resulted in DNA unmethylation percentages among case and control samples of quantitative amplifications with *TNFSF11* unmethylated primers in women and men, respectively. The x-axis details the average of Cq values reported in each reaction. The right y-axis represents the average percent DNA methylation across the *TNFSF11* promoter CpG-rich region. The left y-axis represents the average percent DNA unmethylation across the *TNFSF11* promoter CpG-rich region.

The DNA methylation percentage values were fitted depending on the Cq values. Standard non-linear models resulting for the methylated and unmethylated *TNFSF11* promoter region revealed a significant fitting between the experimental and fundamental values. The resulted standard models allowed us to identify the global DNA methylation percentages in the *TNFSF11* promoter CpG-rich region among otosclerotic cases and controls in women (Figure 3I) and men (Figure 3II) groups. The DNA methylation percentages were assessed as the DNA methylation (M) and DNA unmethylation (U) evaluations using the following formulas, respectively:


$$\begin{array}{r} \begin{array}{c} M\left(\%\right)\;y=ax^{b}\;:\;a=4.296119E15\pm 3.22122E16\;;\\ b=\;-9.8048\pm 2.32352\\ U\left(\%\right)\;100-y\;;\;y=ax^{b\;}\;:a=2.82976E-16\pm 1.58543E\\ -15\;;\\ b=\;12.10925\pm 1.68843 \end{array} \end{array}$$

### Significant Decrease of DNA Methylation Levels of *TNFSF11* Promoter in Otosclerotic Groups

**Figure 4:**
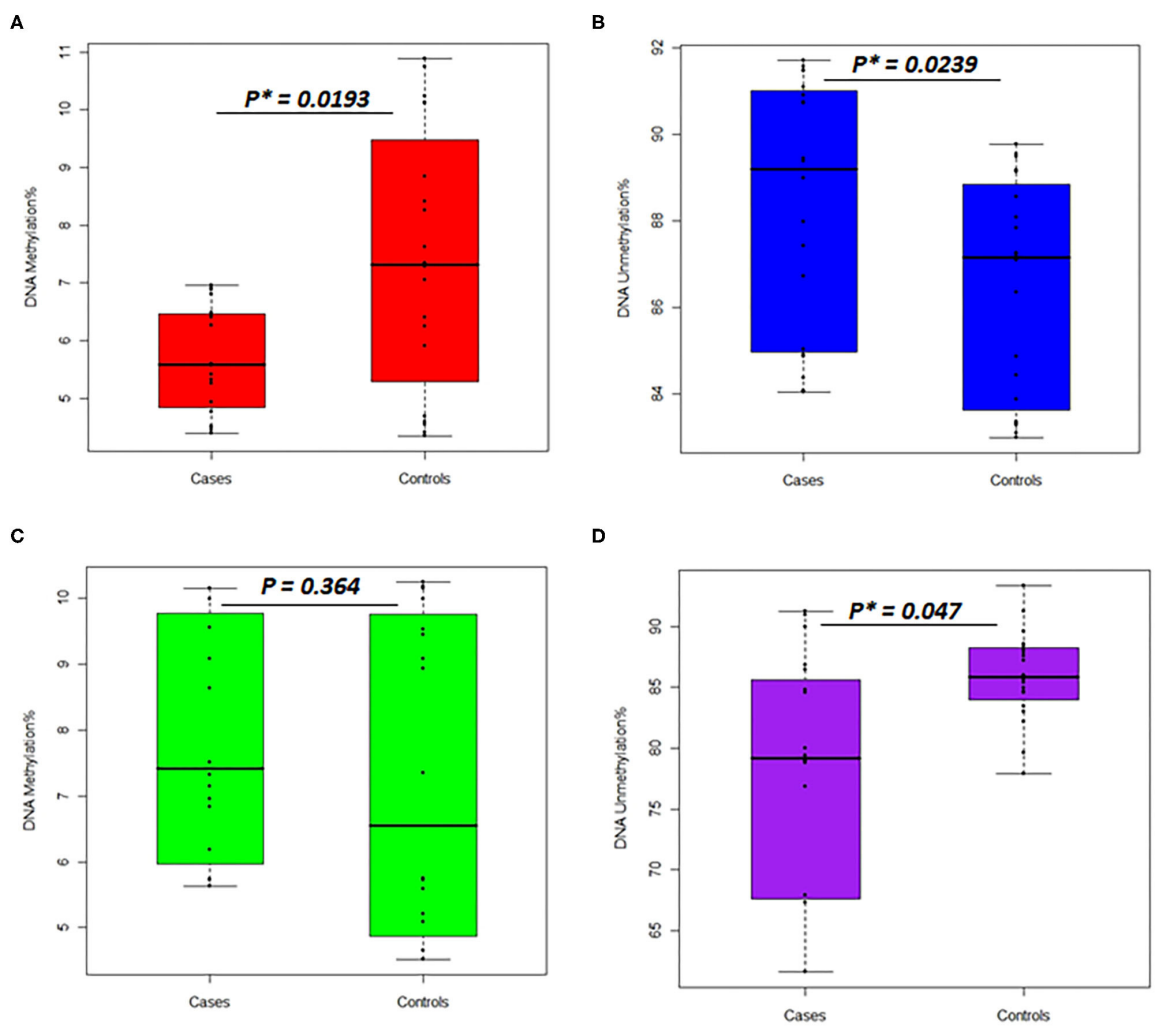
Percentages of DNA methylation and unmethylation in the *TNFSF11* promoter CpG-rich region comparing otosclerotic cases to controls in a Tunisian population. DNA methylation status with methylated *TNFSF11* primers in **(A)** women and **(C)** men. DNA unmethylation status with unmethylated *TNFSF11* primers in **(B)** women and **(D)** men. DNA methylation percentage was calculated as the mean of triplicate Cq values for each sample. Results represent the mean DNA methylation percentage ± SD. For both DNA methylation and unmethylation levels, significant differences were assessed between otosclerotic cases and controls groups with P* <0.05, as evaluated by the Man Whitney-test. The y-axis represents the average percent DNA methylation and DNA unmethylation across the *TNFSF11* promoter CpG-rich region in **(A,C)** and **(B,D)**, respectively.

In the women group, the DNA methylation percentages of the *TNFSF11* promoter CpG-rich region accounted for 5.713 ± 0.924 (average ± standard deviation), 95% CI [5.280–6.145] in the otosclerotic cases group and 7.401 ± 2.253, 95% CI [6.347–8.455] in the controls group, while the DNA unmethylation percentages resulted in 88.35 ± 2.902, 95% CI [86.99–89.70] in the otosclerotic cases group and 86.44 ± 2.519, 95% CI [85.26–87.62] in the controls group. The average values for DNA methylation percentages showed statistically significant differences in both methylation (*p* = 0.0193) (Figure 4A) and unmethylation (*p* = 0.0239) (Figure 4B) status in otosclerotic cases compared to controls.

In the men group, the DNA methylation percentages of the *TNFSF11* promoter CpG-rich region presented 7.825 ± 0.391, 95% CI [7.01–8.64] in the otosclerotic cases group and 7.271 ± 0.533, 95% CI [6.16–8.39] in the controls group, while the DNA unmethylation percentages presented of 76.882 ± 2.351, 95% CI [71.97–81.81] in the otosclerotic cases group and 85.955 ± 0.829, 95% CI [84.22–87.69] in the controls group. The average values for DNA methylation percentages showed statistically significant differences in unmethylation (*p* = 0.047) (Figure 4D) but not in methylation (*p* = 0.364) (Figure 4C) status in OTSC cases with controls taken as a reference. Furthermore, relative DNA methylation analysis showed that the global DNA methylation levels of the *TNFSF11* promoter CpG-rich region in OTSC decreased by 4.53-fold and 4.83-fold as compared to controls in female and male groups, respectively. These DNA hypo-methylation levels may disturb the regulation of the *TNFSF11* gene which could affect the RANK/RANKL/OPG pathway and thus contribute to an increased risk of OTSC.

### Transcription Factor Binding Sites and Regulatory Elements Prediction

Lastly, to evaluate if any specific biological features related to expression regulation are present in the DNA sequence of the *TNFSF11* promoter CpG-rich region, regulatory elements were predicted and correlated to the DNA methylation changes in this CpG-rich region. Interestingly, this latter contains transcription factor binding sites identified in the ORegAnno database and predicted for several highly significant transcription factors by the JASPAR database, including ZNF460, Spi1, EGR1, EGR2, EGR3, EGR4, TFDP1, E2F6, and Plagl1 (Table 2). Additionally, the *TNFSF11* promoter CpG-rich region tends to be DNase-sensitive in 53 different cell types by the ENCODE project with high-scoring clusters (Score = 943, FDR = 0.01), on which chromatin non-condensed is hypersensitive to cutting by the DNaseI enzyme. Hence, the decrease of DNA methylation levels in the *TNFSF11* promoter around TSS in the OTSC samples compared to controls could be correlated and coincided with the high occupancy of regulatory elements that may affect the expression of *TNFSF11* and therefore disturb the RANK/RANKL/OPG pathway.

**Table 2:** Predicted transcription factor binding sites in the *TNFSF11* promoter CpG-rich region.

**Transcription factor**	**Genomic size**	**Score**	***P*-value**
ZNF460	16	627	10^−6^
Spi1	17	606	10^−6^
EGR1	14	644	10^−6^
EGR2	11	632	10^−6^
EGR3	15	716	10^−7^
EGR4	16	643	10^−6^
TFDP1	11	632	10^−6^
E2F6	13	648	10^−6^
Plagl1	13	613	10^−6^

## Discussion

Over the past decades, several studies evidenced the important regulatory role played by the RANK/RANKL/OPG system in osteoclasts maturation, bone modeling, and remodeling. The dynamic equilibrium between the RANKL and OPG levels is critical for maintaining normal bone metabolism and turnover, thus, RANKL/OPG ratio is an important determinant of bone mass and skeletal integrity (57). Alternatively, imbalance in the RANKL/OPG ratio could lead to an altered bone mass (58), increased fractures susceptibility (59), and results in a wide spectrum of metabolic bone diseases, including osteoporosis, rheumatoid arthritis, bone metastases (60), and also OTSC (16).

**Figure 5:**
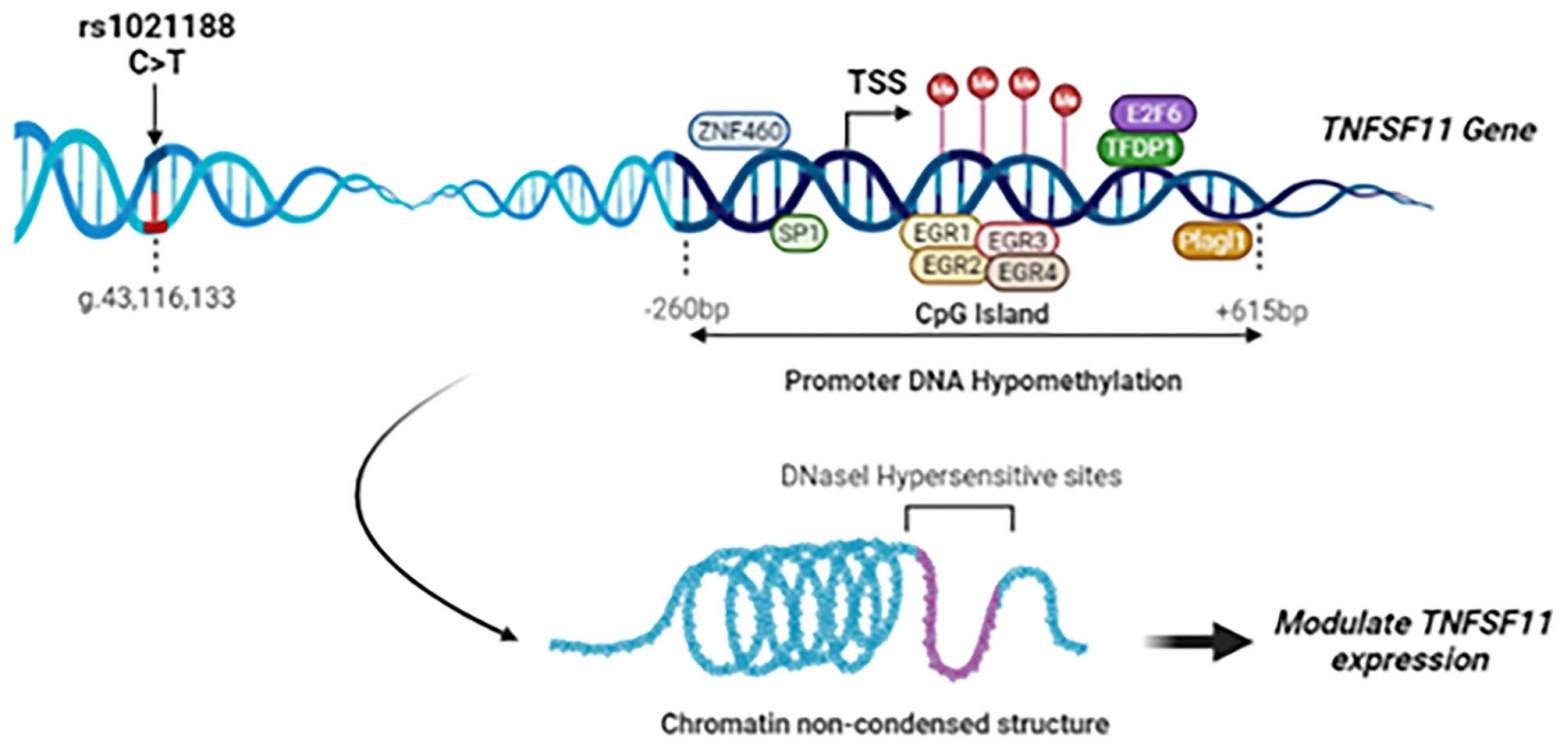
Summary of the study findings: Schematic representation of the rs1021188 SNP, transcription factors binding sites, and CpG island locus in the upstream region of the *TNFSF11* gene (NG_008990.1, Isoform 1). The rs1021188 C/T polymorphism was associated with OTSC in men from a Tunisian North-African ethnic population. The CpG island in the promoter region of the *TNFSF11* gene presents a decrease in the DNA methylation levels in OTSC samples compared to controls. Interaction between promoter DNA methylation surrounding the TSS and transcription factors of the *TNFSF11* gene was predicted: In the DNA hypomethylation, transcription factors can bind DNA allowing RNA polymerase to fix and to start the transcription. Transcription Factors are shown as oval shapes. The *TNFSF11* CpG-rich region was shown to be DNase-sensitive. Chromatin non-condensed permitting transcriptional activation through greater accessibility of transcriptional machinery which may modulate the regulation of *TNFSF11* expression and affect subsequently OTSC pathology.

Given the critical role of the RANK/RANKL/OPG signaling pathway in maintaining/ regulating proper bone metabolism, SNPs in these pathway genes could also be determinants for bone alterations and the onset of pathological conditions. We provided evidence previously that rs3102734 and rs2073618 variants of the *OPG* gene are associated with OTSC in a Tunisian North-African ethnic population (15). To the best of our knowledge, based on a deep literature review and the Mastermind database search, a widely-used bioinformatics platform of a comprehensive genomic association, this is the first study elucidating the roles of genetic association and DNA methylation modifications of *TNFSF11* gene in OTSC (Figure 5).

Herein, we showed an association between the rs1021188 SNP and OTSC in men. It was earlier postulated that the rs1021188 variant of *TNFSF11* was associated with susceptibility of stress fracture injuries in North-American and British elite athletes men (40), which further supports the role of *TNFSF11* in the preservation of bone strength. A GWAS showed that rs1021188 SNP was associated with volumetric cortical bone density (41), and the minor C allele was associated with a significant decrease in bone density, with a notable effect on cortical bone density in men rather than in women (42). *TNFSF11* is controlled by estrogen and other hormones (61, 62). Given that the hormonal status changes extremely during a woman's life (menarche, reproductive period, lactation period, perimenopausal period, and postmenopausal period), the absence of association between the rs1021188 SNP and OTSC in women may be due to the interaction between genetic variation and sex hormones. In another hand, it can be conceivably hypothesized that the *TNFSF11* rs1021188 SNP in itself is not associated with OTSC in women, but probably by combining with other SNPs, the association may become significant. In the same line, Kim et al. reported that an intronic polymorphism of *TNFSF11* (rs2277438) was not associated with bone mineral density in Korean postmenopausal women, but importantly, it was confirmed to interact with the *TNFRSF11B* G1181C SNP to affect bone mineral density (63).

The potential function of the rs1021188 SNP in OTSC is unknown. The significant association between this SNP and OTSC may be explained by the fact that this SNP is in LD with the real causal genetic variation in the *TNFSF11* gene or in a nearby gene. Therefore, genotyping with more variants near this SNP and in the genes involved in the RANKL/RANK/OPG bone remodeling pathway is necessary for future research.

It has been reported that in individuals with osteoporosis, metabolic changes and alteration of bones in the middle ear could be related to conductive hearing loss. Similarities between osteoporosis and OTSC have been reported and the clinical relationship between these two common disorders of bone was supposed when osteoporotic women showed OTSC regardless of age (64). Therefore, significant genetic variations that are associated with hearing loss and osteoporosis, such as the rs1021188 variant, could be an early predictor of either of these two bone disorders. We assume that this SNP is linked to a common mechanism regulating bone metabolism showing an effect of the non-coding variant on clinical phenotypes. This is could be further emphasized by our *in-silico* findings suggesting that the rs1021188 variant may play a role in *TNFSF11* transcription regulation. Since *TNFS11* alterations have established an association with osteoporosis, additional bone mineral density evaluation (such as levels of vitamin D, calcium, phosphorus, and parathyroid hormone) in our patients with OTSC would help us further to ascertain the association between osteoporosis and OTSC. We believe that our findings will serve as a base for futuristic research for both osteoporosis and OTSC disorders.

To avoid selection bias in our association study, it was aimed that the study population was ethnically and genetically homogeneous. Our case-control association study was designed with the matching of Tunisian North-African ethnicity for patients with OTSC and healthy subjects from a local population with the same geographical region (south and southeast of Tunisia) which is in favor to avoid false-positive association results. This is was further confirmed by multivariate analysis using two-way unsupervised hierarchical clustering which showed that the prevalence of rs1021188 SNP is closer to the African populations than to the Dominican populations within the same cluster, albeit, the majority of Dominicans have sub-Saharan African ancestry (65). Nevertheless, the association of rs1021188 in the *TNFSF11* gene needs to be replicated in other populations with different ethnic groups to provide more credibility to this genotype-phenotype association.

We explored the *TNFSF11* SNPs in linkage disequilibrium with the rs1021188 SNP in five populations (American, African, European, South Asian, and East Asian). Euclidean and Ward's linkage method was assessed to evaluate if the rs1021188 SNP was significantly enriched or depleted in each of the studied populations compared with the global average. The *p*-values were then used to create an enrichment/depletion heatmap. Twenty-six loci were identified in LD with rs1021188 exhibited unique allele enrichment/ depletion profiles across the five populations. The European population was highly distinguished from American and African but closer to south Asians that is independent of the other populations. Additionally, the GWAS Catalog (https://www.ebi.ac.uk/gwas/home) showed high levels of linkage disequilibrium (*r*^2^ > 0.8) with rs1021188 SNP in Iberian subpopulation in Spain. This is giving more evidence to the association of rs1021188 SNP with our Tunisian population implying signals of genetic admixture from North-Africa into Iberia populations based on the historical population movements related to both the Muslim conquest and the subsequent *Reconquista* (66).

Considering the possible link between DNA methylation modifications and OTSC; we evaluated the DNA methylation patterns of the CpG-rich region overlapping the TSS in the promoter of *TNFSF11* in OTSC cases and controls for both gender groups. We found a decrease in DNA methylation levels in subjects with OTSC compared to controls in both genders. Consequently, this epigenetic modification by a change in the DNA methylation levels may affect the transcription regulation by an increase in the gene expression of the *TNFSF11* gene as previously documented (47). It has been reported that the tumor-specific shift to transcriptional repression related to DNA methylation at TSSs was confirmed in several tumor types (67). This is also consistent with the fact that DNA methylation affects gene expression by the intervention of transcription factors binding to the corresponding regulatory sites (68). Importantly, we identified that CpG sites within the *TNFSF11* promoter coincide with transcription factor binding sites, including ZNF460, Spi1, EGR1, EGR2, EGR3, EGR4, TFDP1, E2F6, and Plagl1, which seem to play a critical role in transcriptional control of *TNFSF11*. Some of the transcription factors predicted to bind to this CpG-region, such as E2F6, have been linked to modulating bone function (69). Whether these transcription factors target this region requests to be experimentally validated before their potential roles in transcriptional regulation of *TNFSF11* are appraised.

Intriguingly, the studied *TNFSF11* promoter CpG-rich region was predicted to encompass DNase I-Hypersensitive sites. It has been shown that DNase I Hypersensitive sites highly correlate with active genes expression, suggesting these sites can be considered as markers to identify *cis-regulatory* elements associated with the disease (70). This may reflect the fact that DNase I enzyme preferentially target hypomethylated DNA where the molecule is topographically exposed and more reachable to modifying enzymes. It was reported that the methylation status of the CpG locus upstream of the TATA-box moderates the control of cell- and mouse tissue-specific expression of the *TNFSF11* gene and osteoclastogenesis (46). Moreover, our previous finding provided evidence that DNA hyper-methylation of promoter regions in elderly women is correlated with the down-regulation of potential biomarkers in age-related hearing impairment, such as *P2RX2, KCNQ5, ERBB3*, and *SOCS3* (71). The present hypothesis of a coupled DNA hypo-methylation with an increase in the *TNFSF11* gene expression is in line with the study by Priyadarshi et al. (16), that showed increased RANKL-OPG ratio expression in OTSC. Furthermore, elevated RANKL-OPG ratio in children and juvenile serum was associated with increased activation of osteoclasts in skeletal and non-skeletal pathologies (72).

Due to the higher prevalence/risk of OTSC in women which is 2 to 3-fold higher than in men (73), and to avoid gender heterogeneity, the DNA methylation analysis was directed on each gender separately. It has been suggested that hearing disorder is influenced by gender probably through the sex hormones which can affect the bone metabolism of the otic capsule (61). The effect of diverse sex hormones on the ear may affect the labyrinthine function (74). While the RANK/RANKL/OPG system that controls bone metabolism might be regulated by hormones, such as estrogen, progesterone, and prolactin (75). It has been reported that estrogen promotes prolactin release, on one hand, and on the other hand, prolactin increases RANKL and decreases OPG, which influences bone metabolism. This is in favor to consider the effect of hormones on hearing function and the bony otic capsule disorders (61). Furthermore, it is hypothesized that pregnancy is likely to trigger the onset/progress of OTSC during intense periods of hormonal activities (76). Particularly, the variations in these hormone levels during pregnancy may result in low-frequency hearing loss and full feeling of the ear (77). Research on aging has shown that hearing regresses rapidly in post-menopausal women above 50 years of age with poor capacity to hear at low frequencies (78, 79). This may suggest an association effect between *TNFSF11* gene and aging in one hand, and emphasize our findings by the fact that hypomethylation of *TNFSF11* gene is associated with aging phenotype as reported in EWAS Platform, a comprehensive analysis for epigenome-wide association studies (https://ngdc.cncb.ac.cn/ewas).

Considering the abnormal bone remodeling in the human otic capsule which is characterized by irregular bone growth around the stapes footplate and oval window, it is most probably due to a regulatory mechanism for which *TNFSF11* appears to be a key factor. It can be hypothesized that the progression of OTSC is due to local repression of genes involved in bone turnover that affect only the otic capsule and not the general skeleton, like *TNFSF11*.

So far, surgical intervention has remained the gold standard treatment for restoring hearing in patients with even profound conductive hearing loss (80). Non-invasive treatment, however, could not yet be effective for the prevention of OTSC, possibly due to a lack of molecular targets. Understanding of possible cause(s) underlying activation of the triggers in the otospongiotic foci (early stage) during OTSC is required for the evolution of OTSC research and treatments. We believe that identifying candidate transcriptional regulatory elements that control *TNFSF11* expression is of high interest to help in discovering novel bone therapeutic targets for OTSC.

Due to the complexity in accessing and harvesting tissues of the human middle ear (stapes, mucosa, otic capsule…), peripheral blood samples were used in this study as a promising alternative and which was confirmed in other diseases without accessible tissues (81). DNA methylation patterns may depend on specific conditions and change from one tissue type to another. However, it was reported that the DNA methylation levels are well-maintained between tissues and blood cells (82, 83). Moreover, the bone DNA methylation patterns were confirmed as a predictor biomarker of skeletal bone disease progression in peripheral blood samples in an independent cohort of osteoporotic postmenopausal women (84). We are aware of the possible differences of DNA methylation levels in blood samples compared to those of the otic capsule tissues, and it is still a question of practical significance to show that blood might be a rational surrogate for otic capsule tissues when evaluating the effects of DNA methylation patterns.

The most striking finding in our study is that variations in the upstream region of the *TNFSF11* gene, including the association of the rs1021188 SNP with OTSC and the decrease of the DNA methylation levels of *TNFSF11* promoter CpG-rich region in OTSC patients compared to controls, may separately increase the risk of OTSC disease. Together these genetic and epigenetic variations showed that the infrequent genotype CC showed the less range of DNA methylation level and the height range of DNA unmethylation level among all samples compared to CT and TT genotypes (Supplementary Figure 3). As far as, the rs1021188 SNP (C > T) is present at a GC dinucleotide, the frequent T allele is disrupting this GC site, thus, it may be assumed that this could affect DNA methylation regulation of the nearby CpG-rich regions. The relationship between DNA methylation changes and the rs1021188 genotypes in the *TNFSF11* promoter CpG-region could partially explain the reason whether non-coding variants could be associated with the disease.

## Conclusion

In conclusion, we report an association between the *TNFSF11* rs1021188 polymorphism and OTSC in the male gender from a Tunisian North-African population. Linkage disequilibrium analysis showed *TNFSF11* SNPs diversity amongst populations with different ethnicity. In addition, DNA methylation analysis may represent a powerful tool to understand the complexity of OTSC and to determine potent regulatory targets for effective therapies directed for normal bone remodeling. Using bioinformatics analysis, we predicted a potential effect of the rs1021188 SNP and regulatory elements that are correlated with the DNA methylation changes in the *TNFSF11* CpG-rich region and consequently related to transcription regulation. Furthermore, functional analyses on the *TNFSF11* gene will eventually help to define the role of its genetic and epigenetic variations in the OTSC pathology and, hence, to understand the mechanism behind its development.

## Data Availability Statement

The datasets presented in this study can be found in online repositories. The name of the repository and accession number can be found below: Figshare; accession number - 10.6084/m9.figshare.19353518.

## Ethics Statement

The studies involving human participants were reviewed and approved by Regional Committee of the Protection of Persons, Sfax, Tunisia (CPP SUD N°28/2019). Written informed consent was obtained from all participants of this study.

## Author Contributions

AB designed the study, analyzed and interpreted the data, and wrote the manuscript. AC performed the experiments. AT contributed to data interpretation and writing and revision of the manuscript. IA and IC performed the clinical diagnosis of patients. IB and FJ contributed to the data collection of samples. NS, KH, and PVR contributed to the interpretation of the data and revised the manuscript. RH supervised the analysis, revised the manuscript, and provided funding. SM supervised the experimental design, revised the manuscript, and provided funding. All authors contributed to the article and approved the submitted version.

## Funding

This work was supported by the Ministry of Higher Education and Research of Tunisia and by a research grant under the Tunisia and India Agreement on Science and Technology Bilateral Cooperation EPIGENOTOS (to SM and PVR).

## Conflict of Interest

The authors declare that the research was conducted in the absence of any commercial or financial relationships that could be construed as a potential conflict of interest.

## Publisher's Note

All claims expressed in this article are solely those of the authors and do not necessarily represent those of their affiliated organizations, or those of the publisher, the editors and the reviewers. Any product that may be evaluated in this article, or claim that may be made by its manufacturer, is not guaranteed or endorsed by the publisher.

## Glossary

### Abbreviations

ACE: Angiotensin-Converting Enzyme
AGT: AnGioTensinogen
BMP: Bone Morphogenetic Protein
CI: Confidence Interval
COL1A1: Collagen type I Alpha 1
COL1A2: Collagen type I Alpha 2
Cq: Quantification cycle
EGR: Early Growth Response
EWAS: Epigenome-Wide Association Studies
E2F6: E2F Transcription Factor 6
FGF2: Fibroblast Growth Factor 2
FOXL1: Forkhead Box L1
GAPDH: GlycérAldéhyde-3-Phosphate DésHydrogénase
GWAS: Genome-Wide Association Study
IQR: InterQuartile Range;
LD: Linkage Disequilibrium
M-CSF: Macrophage Colony-Stimulating Factor
MEPE: Matrix Extracellular Phosphoglycoprotein
OPG: Osteoprotegerin
OTSC: Otosclerosis
PCR: Polymerase Chain Reaction
Plagl1: PLAG1 Like Zinc Finger 1
qMSP: Quantitative Methylation-Specific Polymerase chain reaction
RANK: Receptor Activator of Nuclear factor Kappa-B
RANKL: Receptor Activator of Nuclear factor Kappa-B ligand
RELN: Reelin
SERPINF1: Serpin family F member 1
SNP: Single Nucleotide Polymorphism
Spi1: Spi-1 Proto-Oncogene
TFDP1: Transcription Factor Dp-1
TGFβ1: Transforming Growth Factor beta 1
TNF: Tumor Necrosis Factor
TNFSF11/TRANCE: TNF Superfamily Member 11
TNFα: Tumor Necrosis Factor alpha
Tm: Melting temperature
TSH2B: H2B Clustered Histone 1
TSS: Transcription Start Site
ZNF460: Zinc Finger Protein 460.
