# Supplementary material for: Genetic Association of rs1021188 and DNA Methylation Signatures of TNFSF11 in the Risk of Conductive Hearing Loss
Source: Front Med (Lausanne). 2022 Apr 18;9:870244. doi: 10.3389/fmed.2022.870244 (PMC9058115; doi:10.3389/fmed.2022.870244)
Supplement: Supplementary file 1 [file Table_1.DOCX]

**Supplementary Table: Frequency Data of rs1021188 SNP C>T across all available populations, extracted From SNP database.**

| **Study** | **Population** | **Group** | **Sample size** | **Ref Allele** | **Alt Allele** | **BioProject ID** | **BioSample ID** |
| --- | --- | --- | --- | --- | --- | --- | --- |
| **TopMed** | Global | Study-wide | 264690 | C=0.227209 | T=0.772791 | PRJNA400167 | |
| **gnomAD - Genomes** | Global | Study-wide | 140130 | C=0.208671 | T=0.791329 | PRJNA398795 | SAMN07488253 |
| **gnomAD - Genomes** | European | Sub | 75918 | C=0.16854 | T=0.83146 | | SAMN10181265 |
| **gnomAD - Genomes** | African | Sub | 41962 | C=0.24577 | T=0.75423 | | SAMN07488254 |
| **gnomAD - Genomes** | American | Sub | 13646 | C=0.31152 | T=0.68848 | | SAMN07488255 |
| **gnomAD - Genomes** | Ashkenazi Jewish | Sub | 3322 | C=0.1863 | T=0.8137 |  | SAMN07488252 |
| **gnomAD - Genomes** | East Asian | Sub | 3130 | C=0.2540 | T=0.7460 |  | SAMN07488251 |
| **gnomAD - Genomes** | Other | Sub | 2152 | C=0.2175 | T=0.7825 |  | SAMN07488248 |
| **The PAGE Study** | Global | Study-wide | 78696 | C=0.26995 | T=0.73005 | PRJNA168052 | SAMN10868975 |
| **The PAGE Study** | AfricanAmerican | Sub | 32516 | C=0.24625 | T=0.75375 | | SAMN10868721 |
| **The PAGE Study** | Mexican | Sub | 10810 | C=0.35809 | T=0.64191 | | SAMN10868735 |
| **The PAGE Study** | Asian | Sub | 8316 | C=0.2929 | T=0.7071 |  | SAMN10868722 |
| **The PAGE Study** | PuertoRican | Sub | 7916 | C=0.2693 | T=0.7307 |  | SAMN10868968 |
| **The PAGE Study** | NativeHawaiian | Sub | 4532 | C=0.2068 | T=0.7932 |  | SAMN10868777 |
| **The PAGE Study** | Cuban | Sub | 4230 | C=0.1986 | T=0.8014 |  | SAMN10868733 |
| **The PAGE Study** | Dominican | Sub | 3828 | C=0.2414 | T=0.7586 |  | SAMN10868734 |
| **The PAGE Study** | CentralAmerican | Sub | 2450 | C=0.3820 | T=0.6180 |  | SAMN10868729 |
| **The PAGE Study** | SouthAmerican | Sub | 1982 | C=0.3522 | T=0.6478 |  | SAMN10868969 |
| **The PAGE Study** | NativeAmerican | Sub | 1260 | C=0.2667 | T=0.7333 |  | SAMN10868739 |
| **The PAGE Study** | SouthAsian | Sub | 856 | C=0.148 | T=0.852 |  | SAMN10868970 |
| **8.3KJPN** | JAPANESE | Study-wide | 16760 | C=0.31169 | T=0.68831 | PRJNA678214 | SAMN16789458 |
| **1000Genomes** | Global | Study-wide | 5008 | C=0.2242 | T=0.7758 | PRJEB6930 | SAMN07490465 |
| **1000Genomes** | African | Sub | 1322 | C=0.2587 | T=0.7413 |  | SAMN07486022 |
| **1000Genomes** | East Asian | Sub | 1008 | C=0.2718 | T=0.7282 |  | SAMN07486024 |
| **1000Genomes** | Europe | Sub | 1006 | C=0.1650 | T=0.8350 |  | SAMN07488239 |
| **1000Genomes** | South Asian | Sub | 978 | C=0.113 | T=0.887 |  | SAMN07486027 |
| **1000Genomes** | American | Sub | 694 | C=0.331 | T=0.669 |  | SAMN07488242 |
| **Genetic variation in the Estonian population** | Estonian | Study-wide | 4480 | C=0.1725 | T=0.8275 | PRJNA489787 | |
| **The Avon Longitudinal Study of Parents and Children** | PARENT AND CHILD COHORT | Study-wide | 3854 | C=0.1723 | T=0.8277 | PRJEB7217 | |
| **UK 10K study - Twins** | TWIN COHORT | Study-wide | 3708 | C=0.1834 | T=0.8166 | PRJEB7218 | |
| **KOREAN population from KRGDB** | KOREAN | Study-wide | 2930 | C=0.3123 | T=0.6877 | PRJNA589833 | |
| **Genome of the Netherlands Release 5** | Genome of the Netherlands | Study-wide | 998 | C=0.176 | T=0.824 | PRJEB5829 | SAMN13000132 |
| **CNV burdens in cranial meningiomas** | Global | Study-wide | 788 | C=0.274 | T=0.726 | PRJEB37584 | SAMN15458720 |
| **CNV burdens in cranial meningiomas** | CRM | Sub | 788 | C=0.274 | T=0.726 |  | SAMN15458720 |
| **Northern Sweden** | ACPOP | Study-wide | 600 | C=0.188 | T=0.812 | PPRJNA503394 | SAMN10359154 |
| **SGDP_PRJ** | Global | Study-wide | 512 | C=0.139 | T=0.861 | PRJNA586841 | |
| **HapMap** | Global | Study-wide | 326 | C=0.221 | T=0.779 | PRJNA60817 | SAMN10820145 |
| **HapMap** | African | Sub | 120 | C=0.250 | T=0.750 |  | SAMN10821181 |
| **HapMap** | American | Sub | 118 | C=0.144 | T=0.856 |  | SAMN10821182 |
| **HapMap** | Asian | Sub | 88 | C=0.28 | T=0.72 |  | SAMN10821184 |
| **Qatari** | Global | Study-wide | 216 | C=0.176 | T=0.824 | PRJNA288297 | SAMN13019808 |
| **A Vietnamese Genetic Variation Database** | Global | Study-wide | 214 | C=0.257 | T=0.743 | PRJNA515199 | SAMN10744005 |
| **Siberian** | Global | Study-wide | 56 | C=0.21 | T=0.79 | PRJNA267856 | SAMN13113809 |
| **The Danish reference pan genome** | Danish | Study-wide | 40 | C=0.23 | T=0.78 | PRJEB7725 | SAMN13003120 |
| **Ancient Sardinia genome-wide 1240k capture data generation and analysis** | Global | Study-wide | 38 | C=0.16 | T=0.84 | PRJEB36033 | SAMN15458807 |

**SUPPLEMENTARY FIGURE CAPTIONS**

**Supplementary Figure 1**: **Regulatory motifs of rs1021188 genotype in the upstream region of *TNFSF11* gene.**

Figure generated using RegulomeDB (version 2.0.3).

**Supplementary Figure 2**: **The rs1021188 SNP effect on RANKL mRNA secondary structures based on the Minimum Free Energy prediction**.

1. Minimum Free Energy plain structure drawing for rs1021188-C. B) Minimum Free Energy plain structure drawing for rs1021188-T. The arrows show the position of the variant with 50 bases upstream (5') and 50 bases downstream (3'). Figures generated using RNAfold Web Server.

**Supplementary Figure 3: Boxplots of DNA methylation and DNA unmethylation levels across the rs1021188 SNP genotypes.**

Boxes display the median and 1-3 interquartile ranges with whiskers that represent the minimum and maximum methylation levels. Dots represent corresponding methylation level data of both cases and controls.
