# Supplementary figures and images for: Genetic Association of rs1021188 and DNA Methylation Signatures of TNFSF11 in the Risk of Conductive Hearing Loss
Source: Front Med (Lausanne). 2022 Apr 18;9:870244. doi: 10.3389/fmed.2022.870244 (PMC9058115; doi:10.3389/fmed.2022.870244)

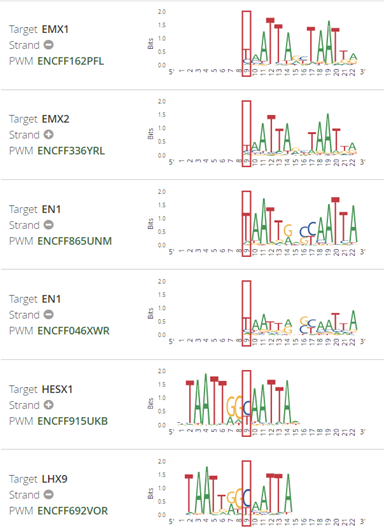

Supplement: Supplementary file 2 [file Image_1.TIF]

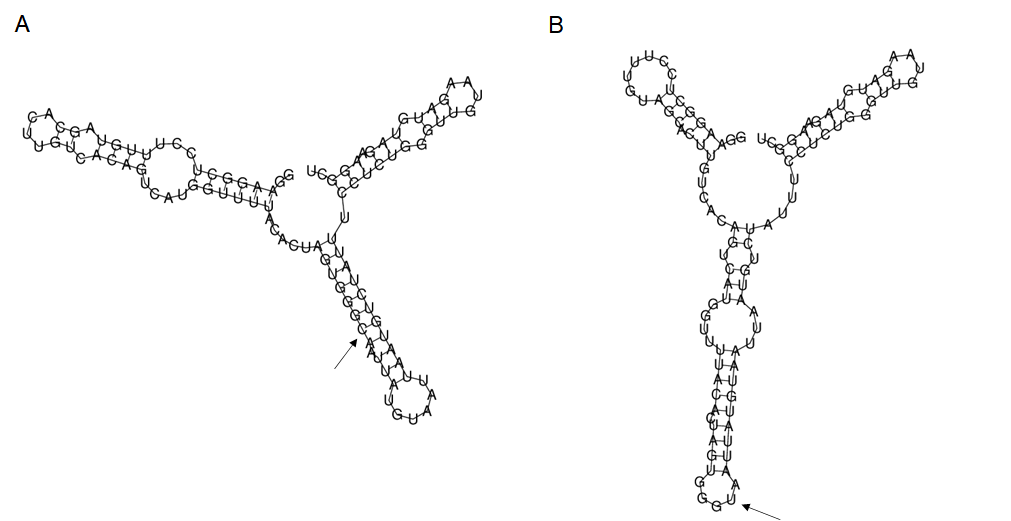

Supplement: Supplementary file 3 [file Image_2.TIF]

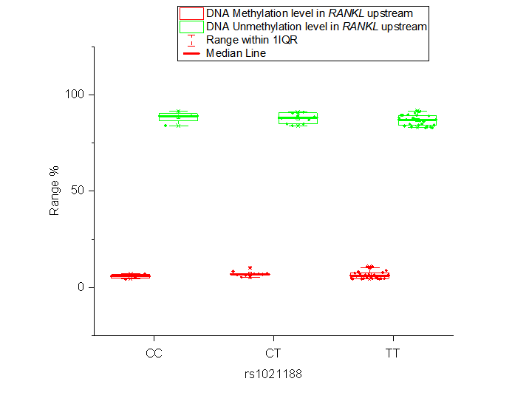

Supplement: Supplementary file 4 [file Image_3.TIF]
